# Comprehensive analysis of ubiquitin‐specific protease 1 reveals its importance in hepatocellular carcinoma

**DOI:** 10.1111/cpr.12908

**Published:** 2020-09-19

**Authors:** Yalei Zhao, Chen Xue, Zhongyang Xie, Xiaoxi Ouyang, Lanjuan Li

**Affiliations:** ^1^ State Key Laboratory for Diagnosis and Treatment of Infectious Diseases The First Affiliated Hospital College of Medicine Zhejiang University Hangzhou China; ^2^ Collaborative Innovation Center for Diagnosis and Treatment of Infectious Diseases Hangzhou China

**Keywords:** deubiquitinases, hepatocellular carcinoma, prognosis, Ubiquitination, ubiquitin‐specific protease 1

## Abstract

**Objectives:**

In this study, we comprehensively analysed the role of ubiquitin‐specific protease 1(USP1) in hepatocellular carcinoma (HCC) using data from a set of public databases.

**Materials and Methods:**

We analysed the mRNA expression of *USP1* in HCC and subgroups of HCC using Oncomine and UALCAN. Survival analysis of *USP1* in HCC was conducted with the Kaplan‐Meier Plotter database. The mutations of USP1 in HCC were analysed using cBioPortal and the Catalogue of Somatic Mutations in Cancer database. Differential genes correlated with *USP1* and WD repeat domain 48 (*WDR48*) were obtained using LinkedOmics. *USP1* was knocked down with small interfering RNA (siRNA) or pharmacologically inhibited by ML‐323 in MHCC97H or SK‐Hep‐1 cell lines for function analysis.

**Results:**

High *USP1* expression predicted unfavourable overall survival in HCC patients. *USP1* showed positive correlations with the abundances of macrophages and neutrophils. We identified 98 differential genes that were positively correlated with both *USP1* and *WDR48*. These genes were mainly involved in the cell cycle, aldosterone synthesis and secretion and oestrogen signalling pathways. Interactions between USP1 and WDR 48 were confirmed using co‐immunoprecipitation. USP1 knockdown or ML‐323 treatment reduced the expression of proliferating cell nuclear antigen (PCNA), cyclin D1 and cyclin E1.

**Conclusions:**

Overall, *USP1* is a promising target for HCC treatment with good prognostic value. *USP1* and *WDR48* function together in regulating cancer cell proliferation via the cell cycle.

## Graphical abstract

Ubiquitin‐specific protease 1 (*USP1*) is significantly overexpressed in hepatocellular carcinoma (HCC). High expression of *USP1* predicts poor survival of HCC patients. In general, *USP1* functions together with its cofactor WD repeat domain 48 (*WDR48*). Here, we find that *WDR48* shows positive correlation with *USP1* in HCC. Ninety‐eight coexpressed genes are mainly involved in the cell cycle, aldosterone synthesis and secretion and oestrogen signalling pathways. USP1 interacts with WDR48 in MHCC97H and SK‐Hep‐1 cells. USP1 knockdown or ML‐323 treatment decreases cell proliferation, and reduces expression of proliferating cell nuclear antigen (PCNA), cyclin D1 and cyclin E1. Overall, *USP1* is a novel therapeutic target for HCC treatment with good prognostic value. *USP1* and *WDR48* function together in regulating cancer cell proliferation via the cell cycle.

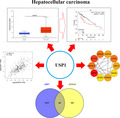

## 1 INTRODUCTION

Primary liver cancer remains a global health challenge with high cancer‐related mortality.^1^ Hepatocellular carcinoma (HCC), the most common primary liver cancer, is the third leading cause of cancer‐related death worldwide.^2^, ^3^ Currently, researchers are focusing on the following aspects: early diagnosis of HCC, prevention of metastasis and recurrence, novel prognostic hallmarks and therapeutic options. However, the therapeutic options for patients with advanced HCC are still limited.^4^ Thus, further understanding the mechanisms of tumorigenesis and progression in HCC is of great interest. In addition, finding new therapeutic targets is still one of the current research priorities.

Ubiquitination, a type of dynamic protein posttranslational modification, is critically involved in various physiological processes.^5^ The dysregulation of ubiquitination leads to several disorders. In recent years, accumulating evidence has revealed the critical role of ubiquitination in tumorigenesis.^6^ In cancer, the effects of ubiquitination are diverse, leading to the suppression or progression of tumorigenic pathways. Components of ubiquitination systems, including the proteasome, ubiquitin, E1/E2/E3 ligases and deubiquitinases, function differently according to their substrates.^7^ Of these, deubiquitinases mediate substrate ubiquitination by removing ubiquitin moieties, thus preventing the degradation of substrate proteins.^8^ In the human genome, more than 100 deubiquitinases are divided into ubiquitin‐specific proteases (USPs), ubiquitin C‐terminal hydrolases, ovarian tumour proteases, Machado‐Joseph disease protein domain proteases and JAB1/MPN/MOV34 metalloenzymes.^9^, ^10^ If their substrates function as tumour suppressors, deubiquitinases prevent their degradation and function as tumour suppressors. However, if their substrates are promoters of tumour progression, deubiquitinases preserve their characteristics and promote tumour progression.^8^, ^11^ Therefore, targeting deubiquitinases has been introduced as a novel therapeutic approach for HCC; however, more data are needed to show the efficacy of this strategy.^7^, ^12^ USPs are cysteine‐dependent proteases and constitute the largest subfamily of deubiquitinases, thus they have gained much interest.^11^ Several high‐quality reviews have summarized the critical roles of USPs in cancer.^10^, ^11^ USP1, a well‐known deubiquitinase, is essential in cellular homoeostasis and the response to DNA damage.^13^, ^14^ As previously reported, USP1 is involved in diverse cellular functions.^15^ USP1 and its cofactor USP1‐associated factor 1, also called WD repeat domain 48 (WDR48), function as regulators in the processes of the DNA damage response, especially in the translation synthesis process and the Fanconi anaemia pathway.^13^, ^16^, ^17^ In general, USP1 and WDR48 form a complex and function together, and WDR48 significantly enhances USP1 activity by stabilizing its expression and mediating its access to substrates.^16^, ^18^ Moreover, USP1 stabilizes inhibitors of DNA binding proteins, which are overexpressed in tumours.^19^, ^20^ USP1 is also involved in the cell cycle. The expression of USP1 is cell cycle dependent, and it reduces the degradation of phosphorylated checkpoint kinase 1 and maintains its activity.^21^ In addition, USP1 is linked to treatment response in cancers. Sourisseau et al reported that USP1 was vital in cis‐diamminedichloroplatinum (II) resistance in non‐small‐cell lung cancer, mainly due to the shortening of the *USP1* mRNA 5’UTR.^14^ Sonego et al demonstrated that USP1 in ovarian cancer cells was linked to the platinum response.^22^ They found that USP1 mediated resistance to platinum by stabilizing Snail and then promoting tumour dissemination.^22^ Overall, USP1 is a promising therapeutic target in cancers. However, the current knowledge about its role in HCC is limited. Thus, determining whether USP1 is pivotal in HCC is of great interest.

In this study, several informatics tools were used to evaluate the expression profile and the prognostic significance of *USP1* in HCC. We explored the correlation between *USP1* expression and immune infiltration. Moreover, we also investigated the underlying mechanisms of *USP1* in HCC by analysing the coexpressed genes of *USP1* and its cofactor *WDR48*. The findings of this study may improve our understanding of *USP1* in HCC.

## 2 MATERIALS AND METHODS

### 2.1 Expression analysis and survival analysis

We searched the Oncomine database (http://www.oncomine.org) with the gene symbol '*USP1'*. The primary filters were set as follows: Analysis type: Differential Analysis; Cancer vs Normal: Liver cancer vs Normal analysis, Hepatocellular Carcinoma vs Normal analysis. Datasets were screened with thresholds of *P*‐value (1E‐4), fold change (2) and gene rank (top 10%). Box plots of the expression data (log_2_ median‐centred intensity) obtained from datasets were generated using GraphPad software. Then, subgroup analysis of the mRNA expression of *USP1* was conducted using the UALCAN database (http://ualcan.path.uab.edu).^23^ The liver hepatocellular carcinoma (LIHC) dataset from The Cancer Genome Atlas (TCGA) was selected for analysis. USP1 expression levels in different subgroups were analysed (sex, age, race, weight, cancer stage, tumour grade, nodal metastasis status and *TP53* mutation status). The promoter methylation levels of *USP1* in HCC and in the subgroups of HCC were also evaluated in comparison with those in normal controls. In addition, we validated the protein expression of *USP1* in the Human Protein Atlas (HPA) database (www.proteinatlas.org).^24^, ^25^ Then, we discovered the prognostic significance of USP1 in HCC using the Kaplan‐Meier Plotter database (http://kmplot.com).^26^

### 2.2 Mutation and immune infiltration analysis

The mutation frequency of *USP1* in HCC was evaluated using cBioPortal (http://www.cbioportal.org/).^27^, ^28^ The mutation types of *USP1* in HCC were further evaluated using the Catalogue of Somatic Mutations in Cancer (COSMIC) database (http://cancer.sanger.ac.uk).^29^, ^30^ We evaluated the correlations between *USP1* expression and immune infiltrates using the Tumor Immune Estimation Resource (TIMER) database (https://cistrome.shinyapps.io/timer/).^31^

### 2.3 Protein‐protein interaction (PPI) network analysis

We employed the LinkedOmics database (http://www.linkedomics.org/login.php) to find differentially expressed genes correlated with *USP1 and WDR 48*.^32^ RNA‐seq data in the TCGA‐LIHC dataset were selected for analysis (Subset: histological type‐hepatocellular carcinoma, n = 371). The correlation coefficients of the differentially expressed genes and *USP1* or *WDR48* were analysed using Spearman tests. The PPI network was constructed using both the STRING database (http://string‐db.org) (interaction score > 0.4) and Cytoscape software (version 3.7.1).^33^, ^34^

### 2.4 Hub gene analysis

To identify the hub genes in the network, we first analysed the clusters of the network with several criteria (degree cut‐off: 2; k‐core: 2; node score cut‐off: 0.2; and max depth: 100). Then, we calculated the node scores using the cytoHubba plug‐in (version 0.1) and ranked the nodes based on degree. Finally, we enriched the hub genes by Gene Ontology (GO) analysis and Kyoto Encyclopedia of Genes and Genomes (KEGG) pathway enrichment analysis in the Database for Annotation, Visualization, and Integrated Discovery (DAVID 6.8, v6.8, https://david.ncifcrf.gov/home.jsp), and the results were visualized with the bioinformatics online tool (http://www.bioinformatics.com.cn). Validation of the correlation between *USP1* and *WDR48* was conducted using the Gene Expression Profiling Interactive Analysis (GEPIA) database (http://gepia.cancer‐pku.cn).^35^

### 2.5 Cell culture, transfection and reagents

The human HCC MHCC97H cell lines were purchased from Guangzhou Cellcook Biotech Co., Ltd. (Cellcook, Guangzhou, China). The human HCC SK‐Hep‐1 cell lines were purchased from Procell Life Science&Technology Co., Ltd. (Procell, Wuhan, China). Both cell lines were cultured in high‐glucose Dulbecco's modified Eagle's medium (DMEM, Gibco, USA), which contained 10% foetal bovine serum, 100 U/mL penicillin and 100 μg/mL streptomycin (Gibco, USA). siRNA‐USP1 (5′‐CCAGAGACAAACUAGAUCA tt‐3′ forward, and 5′‐UGAUCUAGUUUGUCUCUGG tt‐3′ reverse) and non‐targeting control siRNA (NC‐siRNA, 5′‐UUCUCCGAACGUGUCACGUdTdT‐3′ forward, and 5′‐ACGUGACACGUUCGGAGAAdTdT‐3′ reverse) were obtained from Genomeditech Co. Ltd. (Genomeditech, Shanghai, China). And 50 nmol/L siRNA‐USP1 or NC‐siRNA was transfected into MHCC97H or SK‐Hep‐1 cells using INTERFERin (Polyplus transfection, NewYork, USA) according to the manufacturer's protocol. ML‐323 was obtained from MedChemExpress (MCE, China) and dissolved in DMSO. For pharmacological intervention of USP1, ML‐323 was used at 50 μmol/L and cultured for 24 hours (h), and 0.1% DMSO was used as vehicle control.

### 2.6 Cell viability

MHCC97H and SK‐Hep‐1 were seeded in the 96‐well plates with 5000 cells/well and incubated for overnight. The cells were transfected with siRNA‐USP1 or NC‐siRNA for 48 hours. Then, cell viability was analysed using cell counting kit‐8 (CCK‐8, APExBIO, USA) according to the manufacturer's protocol. Briefly, fresh medium was changed after transfection and 10 μL CCK‐8 was added to each well. After incubation at 37°C for 4 hours, the absorbance at 450 nm was obtained using an Epoch 2 microplate reader (BIOTEK).

### 2.7 Western blotting analysis and co‐immunoprecipitation

After 48‐hour transfection or 24‐hour pharmacological intervention, cell lysates were obtained using RIPA lysis buffer (GenStar, Shenzhen, China) with protease inhibitor (Beyotime Biotechnology, Shanghai, China). Total protein was extracted, and the concentration was quantified using BCA kits (Thermo Scientific, USA). Subsequently, 20‐25 μg sample protein was separated by 4%‐20% Sure‐PAGE gels (GenScript, Nanjing, China) and transferred to polyvinylidene fluoride (PVDF) membranes. The primary antibodies against USP1 and WDR48 were obtained from Proteintech group (Proteintech, China). The primary antibodies against cyclin D1, cyclin E1 and glyceraldehyde‐3‐phosphate dehydrogenase (GAPDH) were purchased from Cell Signaling Technology (CST, USA). PVDF membranes were blocked with Quickblock™ buffer (Beyotime Biotechnology, Shanghai, China) and incubated with primary antibodies at 4°C overnight. Next day, PVDF membranes were rinsed and incubated with secondary antibodies (Abcam, USA) at room temperature for 1 hour, and then were visualized by chemiluminescence reagents (Beyotime Biotechnology, Shanghai, China). For co‐immunoprecipitation, we used an IP/COIP kit from Absin (Absin, Shanghai, China). COIP was conducted according to the manufacturer's protocol. Briefly, cells were lysed and incubated on ice, then centrifuged at 14 000 g for 10 minutes at 4°C. The supernatant was incubated with protein A/G agarose beads for pre‐clean. Subsequently, it was immunoprecipitated with an antibody against USP1 (Proteintech, China) or normal rabbit IgG (CST, USA) overnight at 4°C. Next day, the immunoprecipitated complexes were incubated with protein A/G agarose beads for 1 hour at 4°C. After incubation, the immunoprecipitated complexes were rinsed and analysed by Western blotting. Input was used as positive control.

### 2.8 Quantitative real‐time PCR (qRT‐PCR)

For MHCC97H and SK‐Hep‐1 cells, total RNA was isolated using a RNA fast 200 kit (Fastagen, Shanghai, China). Complementary DNA was obtained using PrimeScript™ RT Master Mix (Takara, Shiga, Japan). For qRT‐PCR, the following primers were used: human USP1, 5′‐GCTGCTAGTGGTTTGGAGTTT‐3′ (Forward) and 5′‐GCATCACAACCGCAAATAATCC‐3′ (Reverse); human WDR48, 5′‐AGAAGTACAACCGAAATGGAGTC‐3′ (Forward) and 5′‐ACAATGTCGTTTACCCAATCAGT‐3′ (Reverse); human GAPDH, 5′‐GGAGCGAGATCCCTCCAAAAT‐3′ (Forward) and 5′‐GGCTGTTGTCATACTTCTCATGG‐3′ (Reverse). Relative expression of USP1 and WDR48 were normalized to GAPDH and were analysed using the 2^−ΔΔCT^ method.

## 3 RESULTS

### 3.1 High expression of USP1 in HCC

**Figure 1:**
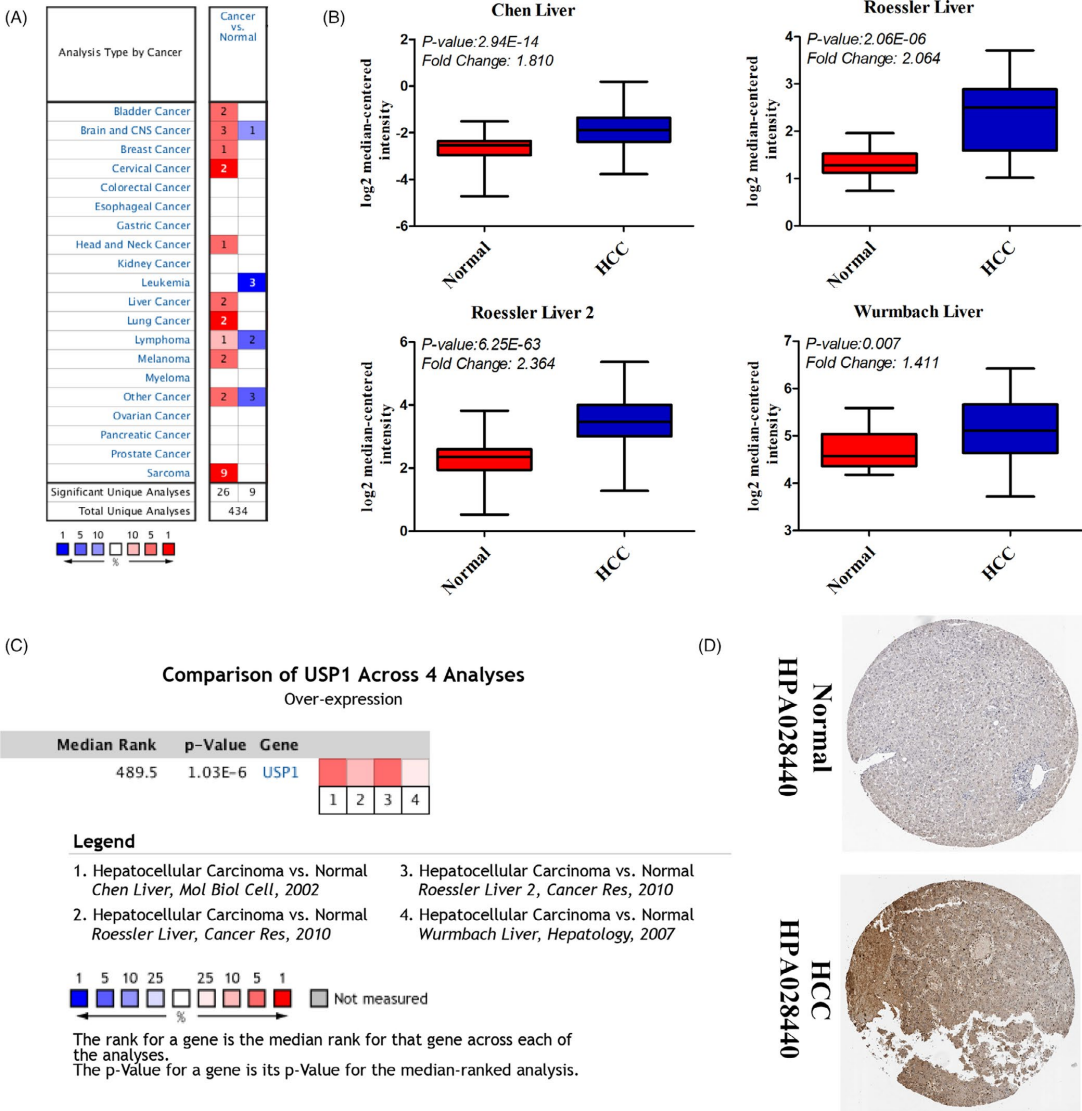
The elevated mRNA expression of *USP1* in hepatocellular carcinoma (HCC). A, *USP1* is overexpressed in several types of cancers (Oncomine database) (Cancer vs normal: overexpression—red colour, downexpression—blue colour). B, *USP1* is overexpressed in hepatocellular carcinoma (HCC) (Oncomine database, Chen liver, Roessler liver, Roessler liver 2 and Wurmbach liver). C, Comparison of *USP1* across 4 studies (Ocomine database). D, Protein expression of USP1 is elevated in HCC (The Human Protein Atlas database)

**Figure 2:**
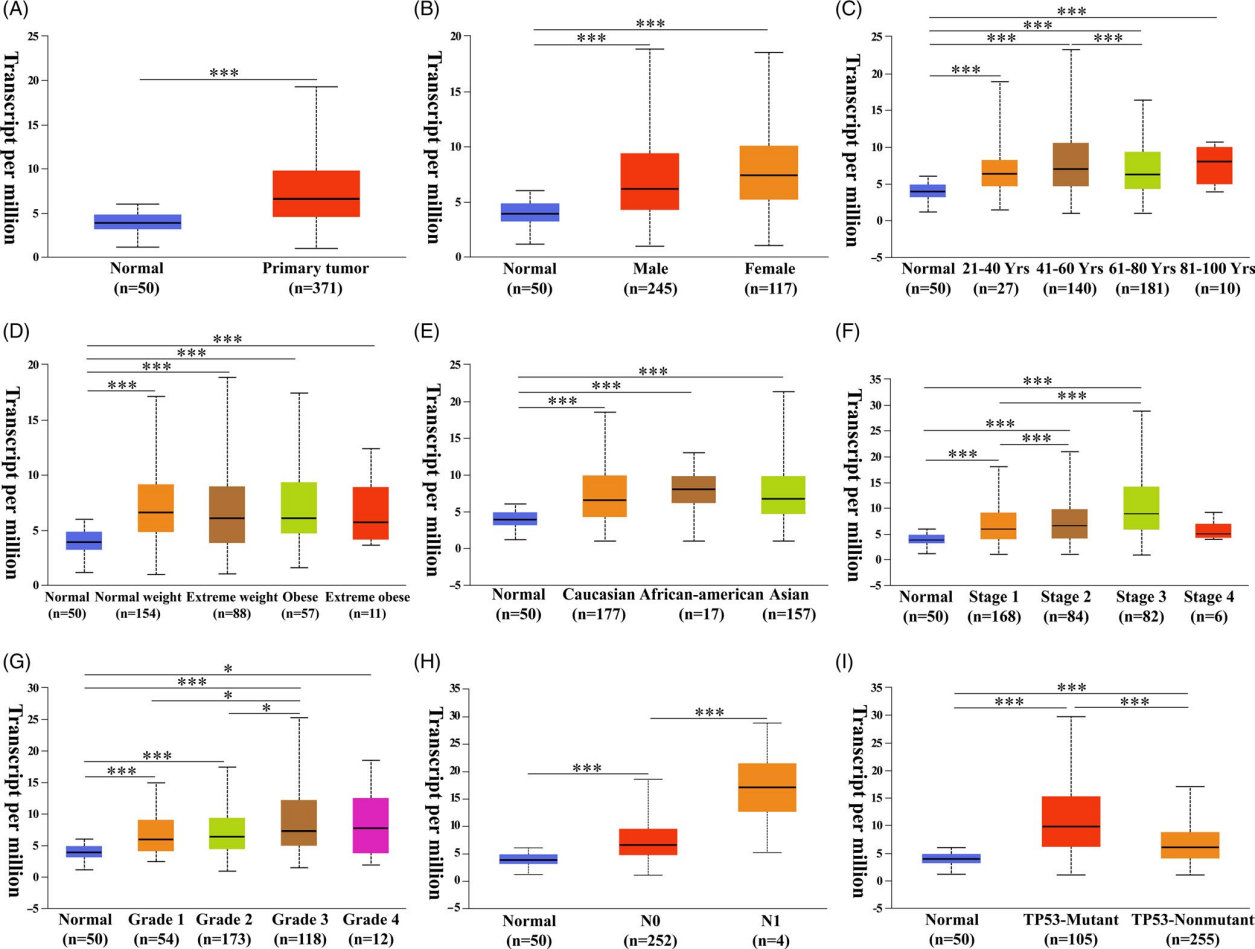
Subgroup expression analysis of *USP1* in HCC. A, mRNA expression of *USP1* in normal and HCC patients. B‐E, *USP1* mRNA expression levels of HCC patients in subgroups with different genders, ages, weights and races). F‐G, *USP1* mRNA expression levels of HCC patients with different tumour stages and tumour grades. H, *USP1* mRNA expression levels of HCC patients with different metastasis status. I *USP1* mRNA expression levels of HCC patients with *TP‐53* mutant or *TP‐53* non‐mutant. A‐I, Graphs are generated from the UALCAN database, *P < .05, **P < .01, ***P < .001

After data mining in the Oncomine database, we found that the mRNA expression of *USP1* was elevated in various types of cancers (cancer vs normal), such as liver cancer, sarcoma and bladder cancer (Figure 1A). Then, we further focused on its expression in HCC, which is the most prevalent primary liver cancer. Data from 4 datasets (Roessler liver 2, Roessler liver, Chen liver and Wurmbach liver) were selected (Figure 1B).^36^, ^37^, ^38^ We performed a meta‐analysis of *USP1* expression in the 4 studies with the following thresholds: p‐value (1E‐4), fold change (2) and gene rank (top 10%) (Figure 1C). All of the results showed that *USP1* was significantly upregulated in HCC tissues compared with normal tissues (*P* < .05). In the Roessler liver 2, Roessler liver, Chen liver and Wurmbach liver datasets, *USP1* showed 2.364‐fold, 2.064‐fold, 1.810‐fold and 1.411‐fold increases in HCC, respectively (Figure 1B). Moreover, *USP1* protein expression was analysed using the HPA database. We found that most types of cancers displayed *USP1* positive staining. Image of normal liver (Patient IDs: 2429) and image of HCC liver (Patient IDs: 2556) are presented here. USP1 was not detected in normal liver and showed weak to medium staining in HCC liver (Figure 1D). To increase the reliability of the results, we further validated the significant overexpression of *USP1* in LIHC data from TCGA using the UALCAN database. As shown in Figure 2A, the mRNA expression of *USP1* was elevated in the LIHC samples (n = 371) compared with the normal samples (n = 50). Subgroup analysis showed that *USP1* was also upregulated in different subgroups of HCC, including the subgroups of sex, age, race and weight (Figure 2B‐E). Regarding cancer stage and tumour grade, we found that *USP1* was overexpressed in stages 1‐3 and grades 1‐4 (Figure 2F,G). In addition, *USP1* was overexpressed in HCC patients without regional lymph node metastasis but not in patients with metastasis (Figure 2H). *USP1* showed a positive association with *TP53* mutation status and was significantly overexpressed in HCC patients with *TP53* mutations (Figure 2I). We also evaluated the promoter methylation level of *USP1* in LIHC; however, no significance was found between LIHC and normal samples (Figure S1). Taken together, these results indicated that the high expression of *USP1* was closely associated with tumour progression.

### 3.2 The prognostic significance of *USP1* in HCC patients

**Figure 3:**
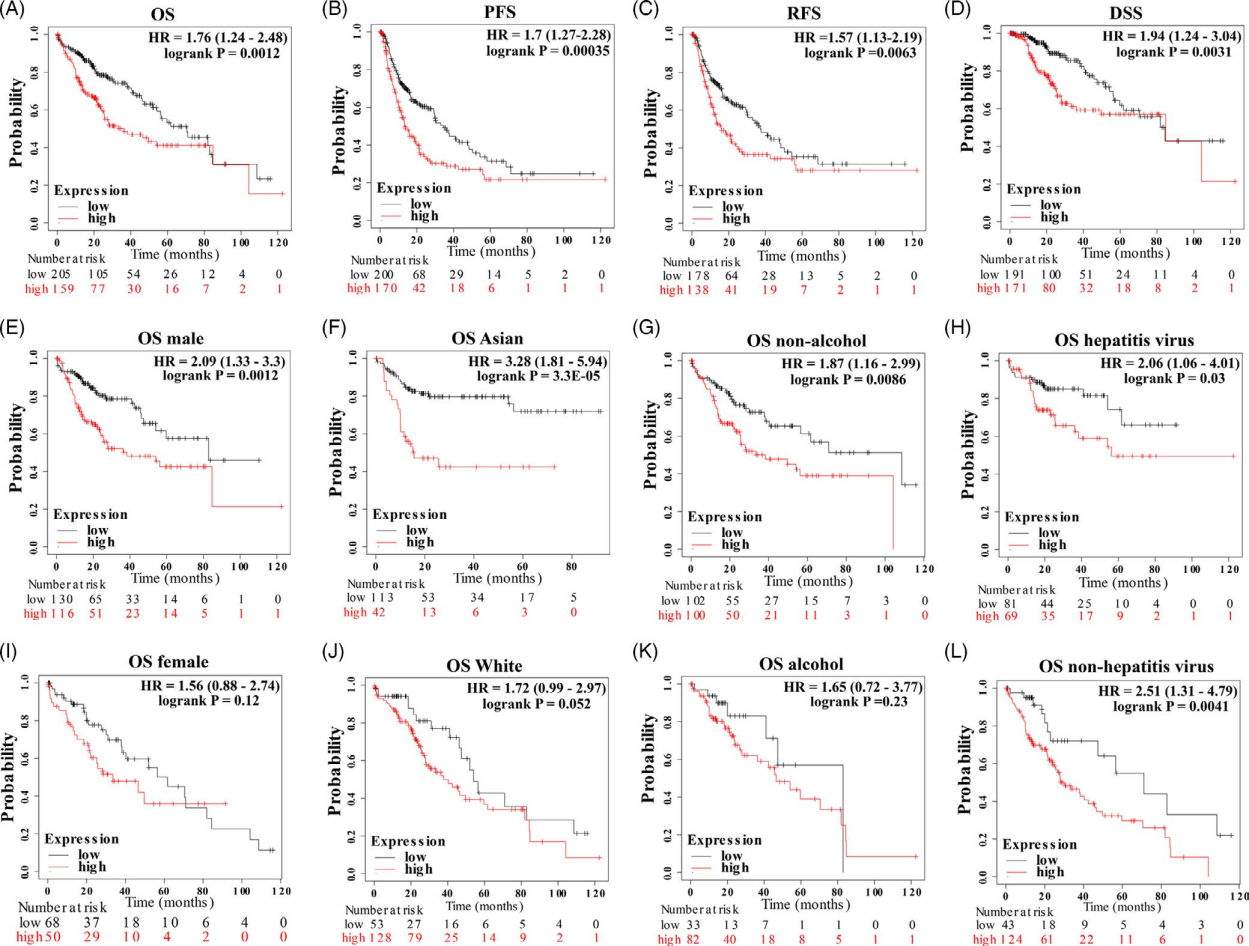
High expression of *USP1* predicts poor prognosis in HCC. A‐L, Graphs generated from the Kaplan‐Meier Plotter database show the prognostic values of *USP1* in HCC patients. OS, overall survival; RFS, relapse‐free survival; PFS, progression‐free survival; DSS, disease‐specific survival; HR, hazard ratio

Thus, we postulated whether *USP1* could function as a prognostic hallmark of HCC patients. Using the Kaplan‐Meier Plotter database (dividing the patients by the auto‐selected best cut‐off), we evaluated the prognostic significance of *USP1* in HCC patients (n = 364). High *USP1* expression was associated with poor overall survival (OS, HR = 1.76 (1.24‐2.48), log‐rank *P* = .0012), relapse‐free survival (RFS, HR = 1.57 (1.13‐2.19), log‐rank *P* = .0063), progression‐free survival (PFS, HR = 1.7 (1.27‐2.28), log‐rank *P* = .00035) and disease‐specific survival (DSS, HR = 1.94 (1.24‐3.04), log‐rank *P* = .0031) of HCC patients (Figure 3A‐D). In addition, high *USP1* expression was also associated with poor OS of HCC patients who were male, Asian and non‐alcohol consumed (Figure 3E‐G), but not those patients who were female, White race and alcohol consumed (Figure 3I‐K). For patients who were hepatitis virus infected or non‐hepatitis virus infected, high *USP1* expression predicted their poor survival (Figure 3H,L). In conclusion, high *USP1* expression was associated with poor prognosis of HCC patients.

### 3.3 Mutations of *USP1* in HCC

**Figure 4:**
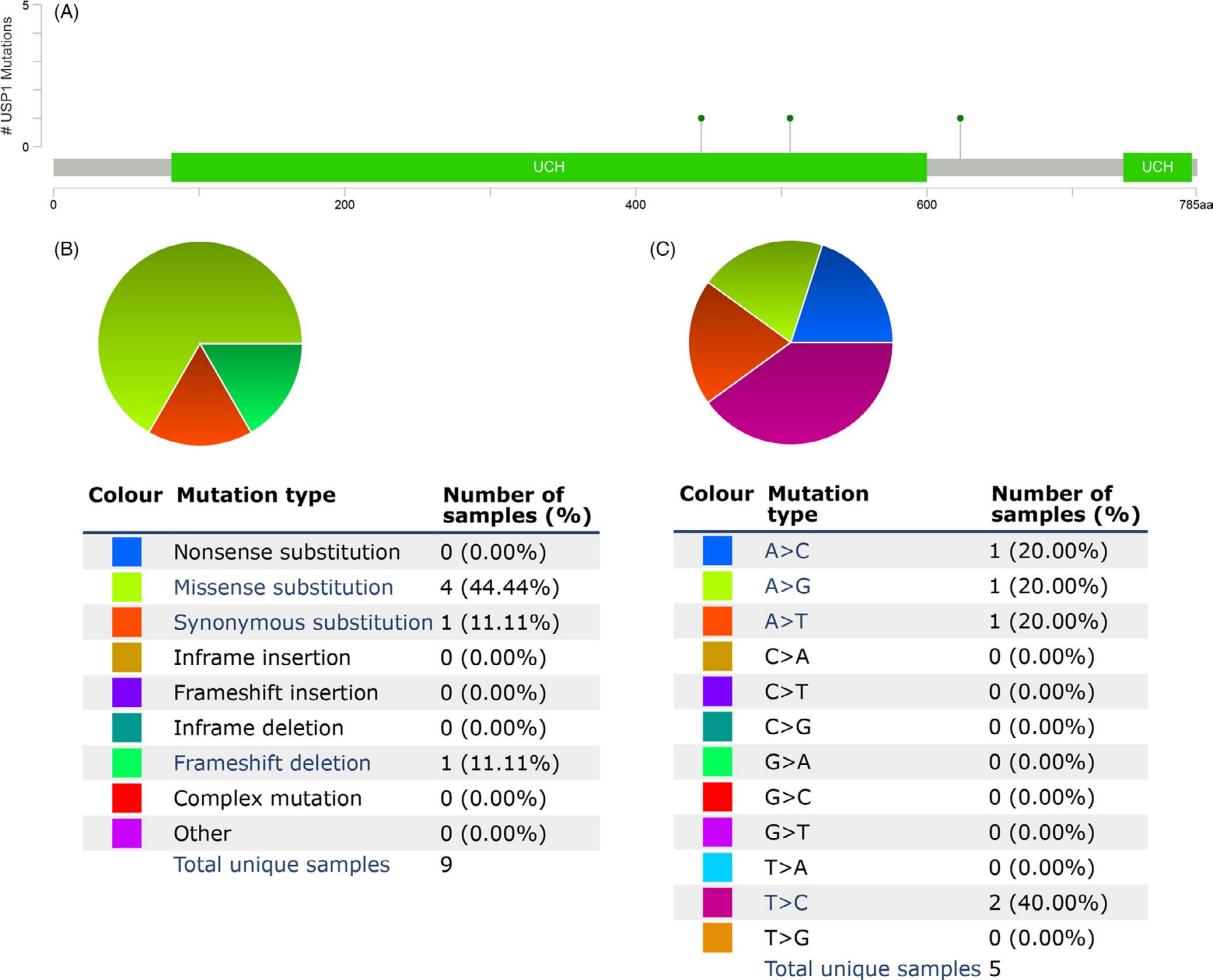
*USP1* mutations in HCC. A, The schematic representation of USP1 mutations in HCC (cBioPortal). B‐C, The mutation types of AGRN (%) in HCC the Catalogue of Somatic Mutations in Cancer (COSMIC) database

The mutation frequency of *USP1* in HCC was evaluated in the cBioPortal database. Five datasets (MSK, AMC, INSERM, RIKEN and TCGA‐PanCancer Atlas), which included 1000 samples, were selected for analysis.^39^, ^40^, ^41^, ^42^, ^43^ The somatic mutation frequency of *USP1* in HCC was 0.3%, which mainly consisted of missense mutations (Figure 4A). This mutation frequency was relatively low, only 3 in 1000 samples. Therefore, we failed to find a relationship between *USP1* mutation and the prognosis of HCC patients (Figure S2). Furthermore, the mutation types of *USP1* were further evaluated in another database, COSMIC. For clarity, two pie charts of the mutation types are shown in Figure 4. Missense substitutions occurred in approximately 44.44% of the samples, synonymous substitutions occurred in 11.11% of the samples, and frameshift deletions occurred in 11.11% of the samples (Figure 4B). The substitution mutations mainly occurred at T > C (40.00%), followed by A > C (20.00%), A > G (20.00%) and A > T (20.00%) (Figure 4C).

### 3.4 The association of *USP1* expression and immune infiltration in HCC

**Figure 5:**
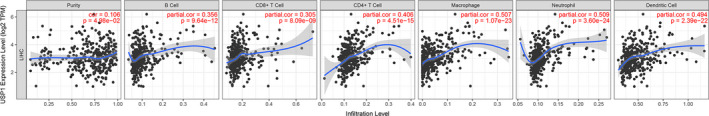
*USP1* is associated with immune infiltration in HCC. Graphs generated from Tumor Immune Estimation Resource database (TIMER) show the correlations between *USP1* and immune cell infiltrations

We investigated the association of *USP1* expression and immune infiltration using the TIMER database. The correlation coefficients between *USP1* expression and the abundances of six immune infiltrates (B cells, CD8 + T cells, CD4 + T cells, macrophages, neutrophils and dendritic cells) were analysed using Spearman tests (tumour purity adjusted). We found that *USP1* expression had a slightly positive correlation with tumour purity (R = 0.106, *P* = 4.98E‐02). Moreover, *USP1* expression had significant positive correlations with all six immune infiltrates, especially neutrophils (*r* = .509, *P* = 3.60E‐24) and macrophages (*r* = .507, *P* = 1.07E‐23) (Figure 5). In addition, we also analysed the correlations between *USP1* expression and related immune cell gene markers. Correlation coefficients were adjusted by tumour purity. Consistent with the above results, *USP1* had significant positive correlations with almost all the selected gene markers of immune cells. Of these, the top five gene markers were *GATA3* (*r* = .612), *CCR8* (*r* = .559), *STAT5B* (*r* = .535), *BDCA‐4* (*r* = .508) and *STAT1* (*r* = .496) (Table 1). Taken together, these results suggest that *USP1* is critically involved in immune infiltration during the progression of HCC.

**Table 1:** Correlations between *USP1* and immune cells’ gene markers in HCC

Immune cell types	Markers	Non‐adjusted		Purity‐adjusted	
		Correlation	*P*‐value	Correlation	*P*‐value
CD8 + T cell	*CD8A*	.202	.000	.285	.000
	*CD8B*	.144	.007	.208	.000
T cell (general)	*CD3D*	.146	.006	.217	.000
	*CD3E*	.146	.007	.249	.000
	*CD2*	.150	.005	.241	.000
Th1	*T‐bet (TBX21)*	.134	.013	.201	.000
	*STAT4*	.237	.000	.275	.000
	*STAT1*	.463	.000	.496	.000
	*IFN‐r*	.215	.000	.260	.000
	*TNF‐a*	.331	.000	.419	.000
Th2	*GATA3 (QRSL1)*	.608	.000	.612	.000
	*STAT6*	.325	.000	.327	.000
	*STAT5A*	.429	.000	.473	.000
	*IL13*	.111	.040	.113	.037
Th17	*STAT3*	.427	.000	.467	.000
	*IL17A*	.085	.114	.090	.095
Treg	*FOXP3*	.321	.000	.355	.000
	*CCR8*	.493	.000	.559	.000
	*STAT5B*	.543	.000	.535	.000
	*TGFb*	.272	.000	.347	.000
B cell	*CD19*	.192	.000	.243	.000
	*CD79A*	.120	.026	.197	.000
TAM	*CCL2*	.198	.000	.299	.000
	*CD68*	.247	.000	.328	.000
	*IL10*	.316	.000	.418	.000
M1 Macrophage	*INOS (NOS2)*	.115	.032	.126	.019
	*IRF5*	.397	.000	.399	.000
	*COX2(PTGS2)*	.283	.000	.391	.000
M2 Macrophage	*CD163*	.311	.000	.415	.000
	*VSIG4*	.269	.000	.370	.000
	*MS4A4A*	.280	.000	.392	.000
Neutrophil	*CD66b (CEACAM8)*	.099	.067	.111	.039
	*CD11b (ITGAM)*	.396	.000	.459	.000
	*CCR7*	.126	.019	.222	.000
Dendritic cell	*HLA‐DPB1*	.222	.000	.316	.000
	*HLA‐DQB1*	.157	.003	.232	.000
	*HLA‐DRA*	.295	.000	.396	.000
	*HLA‐DPA1*	.274	.000	.374	.000
	*BDCA‐1 (CD1C)*	.168	.002	.237	.000
	*BDCA‐4 (NRP1)*	.475	.000	.508	.000
	*CD11c (ITGAX)*	.351	.000	.454	.000

### 3.5 High *WDR48* expression correlated with *USP1* and predicted unfavourable prognosis in HCC patients

**Figure 6:**
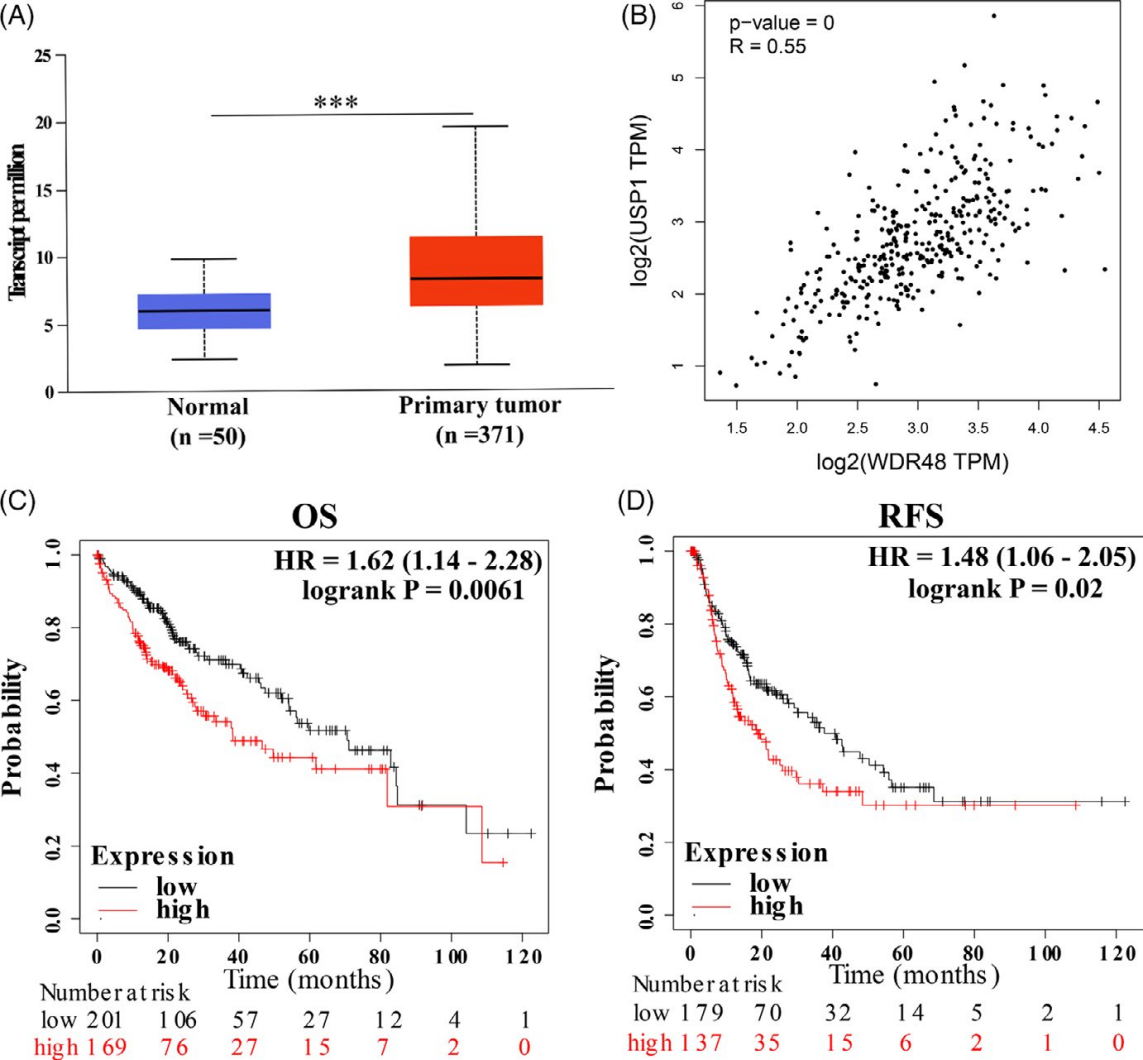
*WDR48* is overexpressed in HCC and predicts poor prognosis. A, *WDR48* mRNA express is overexpressed in HCC (UALCAN). B, *WDR48* is positively correlated with *USP1* in HCC (the Gene Expression Profiling Interactive Analysis database, GEPIA). C‐D, High *WDR48* expression predicts poor OS and RFS in HCC. HR: hazard ratio

To reveal the role of *WDR48*, the cofactor of *USP1*, in HCC, we evaluated its expression and prognostic significance. Using the UALCAN database, we found that *WDR48* was also significantly overexpressed in HCC (Figure 6A). Consistent with our knowledge, its expression in HCC was positively correlated with *USP1* (GEPIA: *r* = .55, *P*‐value = 0, Figure 6B). Intriguingly, we found that high *WDR48* expression was also associated with poor OS and RFS in HCC patients (Figure 6C,D).

### 3.6 Differential genes correlated with both *USP1* and *WDR48* in HCC

**Figure 7:**
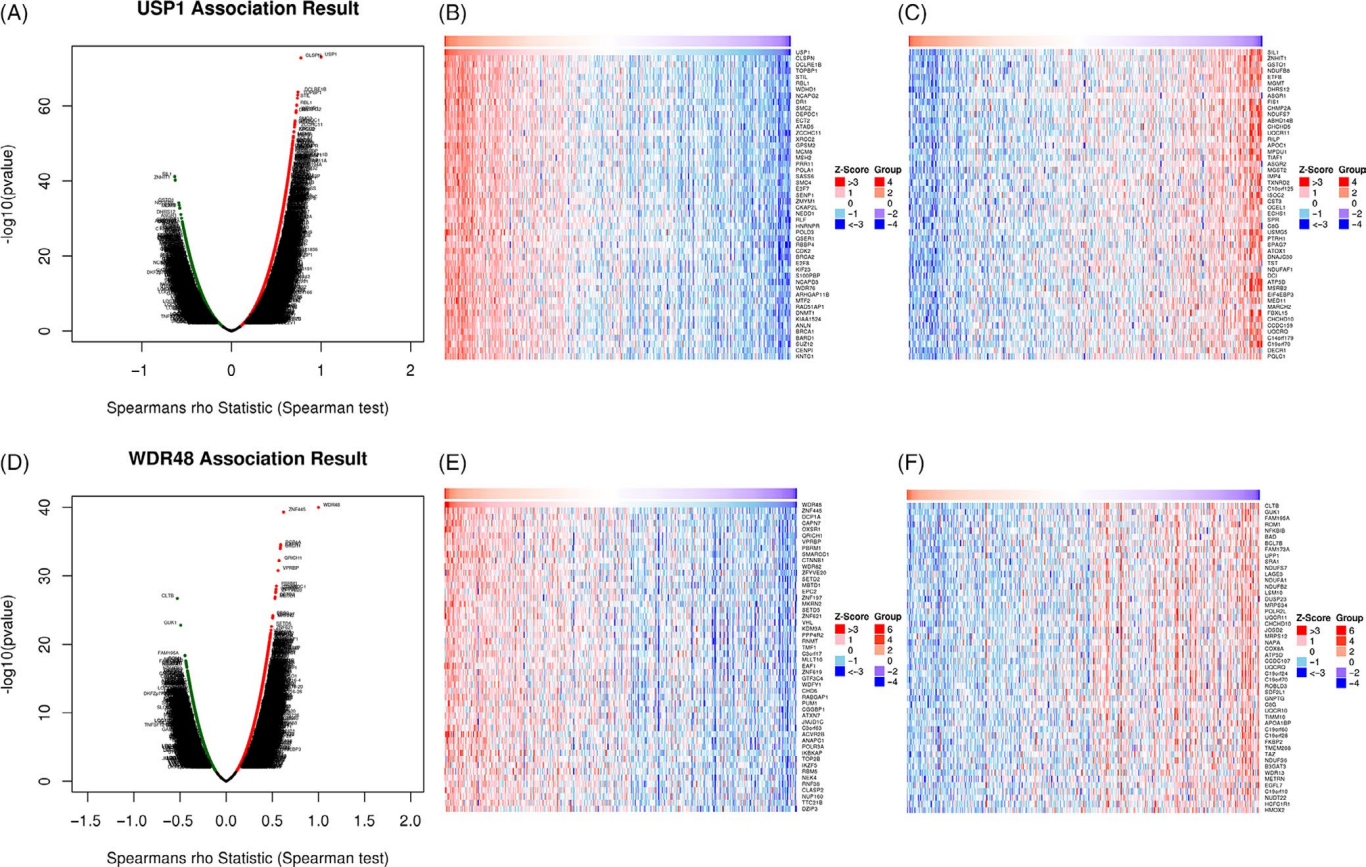
Differentially expressed genes that correlated with *USP1* or *WDR48* in HCC. A, Correlations between *USP1* and differently expressed genes (Spearman correlation analysis). B‐C, Heat maps show the genes that are positively or negatively correlated with *USP1* (Top 50 genes are shown). D, Correlations between *WDR48* and differently expressed genes (Spearman correlation analysis). E‐F, Heat maps show the genes that are positively or negatively correlated with *WDR48* (Top 50 genes are shown)

**Figure 8:**
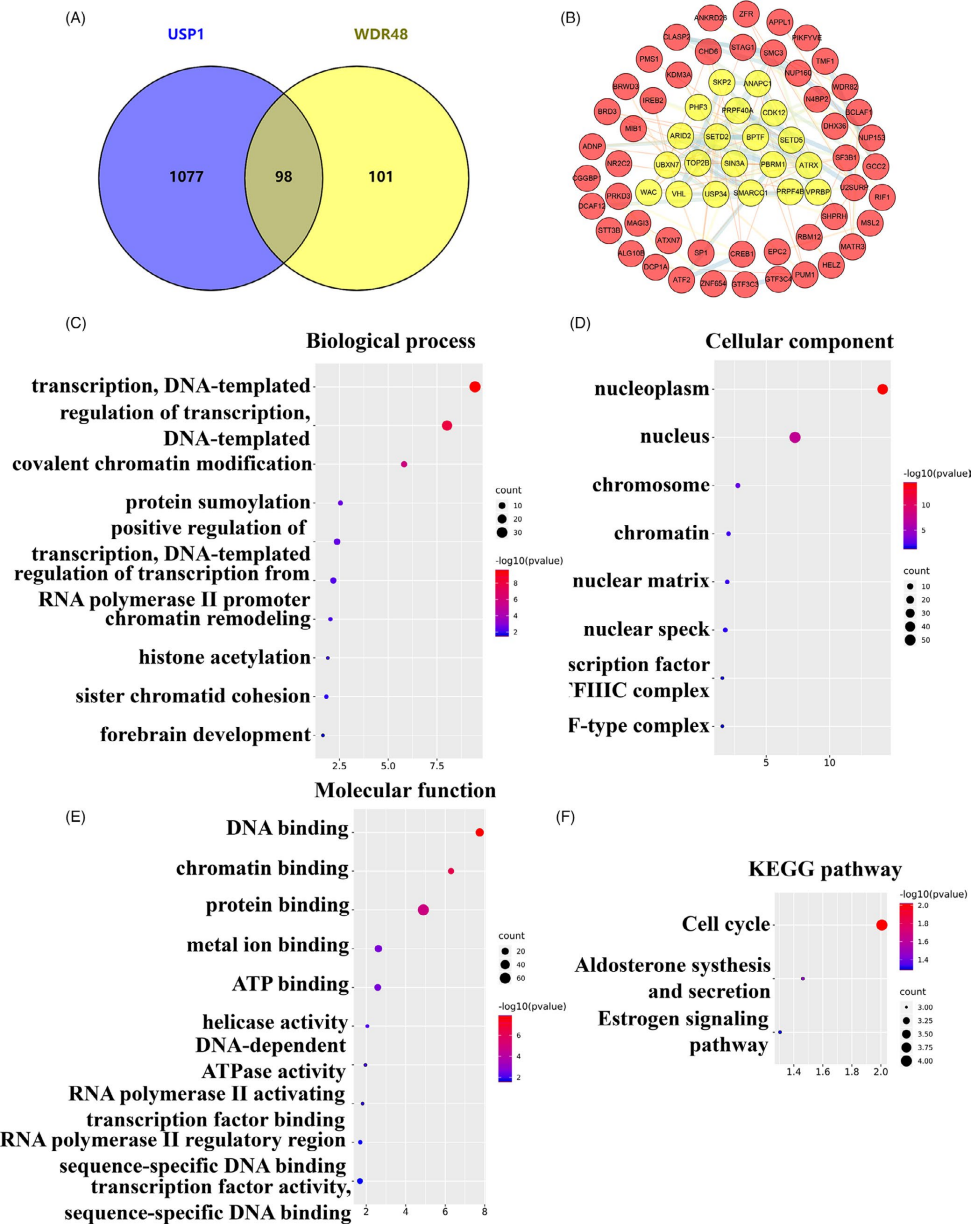
Functional analysis of genes positively correlated with both *USP1* and *WDR48*. A, The venn results show that 98 genes are positively correlated with both *USP1* and *WDR48*. B, The interaction network of the 98 genes and the most important clusters are shown in yellow colour. C‐F, GO analysis and KEGG enrichment of the 98 genes

The LinkedOmics database was used to identify differentially expressed genes that were correlated with *USP1* and *WDR48* in HCC. Based on the Spearman test, the differentially expressed genes correlated with *USP1* and *WDR48* were identified (Figure 7A,D). The top 50 positively (*r* > 0) and top 50 negatively (*r* < 0) correlated genes are shown in heat maps (Figure 7B,C,E,F). Based on the Spearman test, we selected the positively correlated genes with coefficient > 0.4. Finally, 1175 genes positively correlated with *USP1* and 199 genes positively correlated with *WDR48* were selected. Among these, 98 genes showed positive correlations with both *USP1* and *WDR48*, and these genes were selected for further analysis (Figure 8A). The 98 differentially expressed genes were input into STRING and Cytoscape to construct a PPI network (Figure 8B) and were used for GO and KEGG enrichment analysis using DAVID. The following biological processes were significantly affected: transcription, regulation of transcription, covalent chromatin modification, etc (Figure 8C). The cellular component terms were mainly enriched in the nucleoplasm, nucleus, centrosome, etc (Figure 8D). The molecular function terms were mainly enriched in DNA binding, chromatin binding, protein binding, etc (Figure 8E). The KEGG results showed that the coexpressed genes were mainly involved in the cell cycle, aldosterone synthesis and secretion and oestrogen signalling pathways (Figure 8F).

### 3.7 Identification of hub genes and their prognostic value in HCC

**Figure 9:**
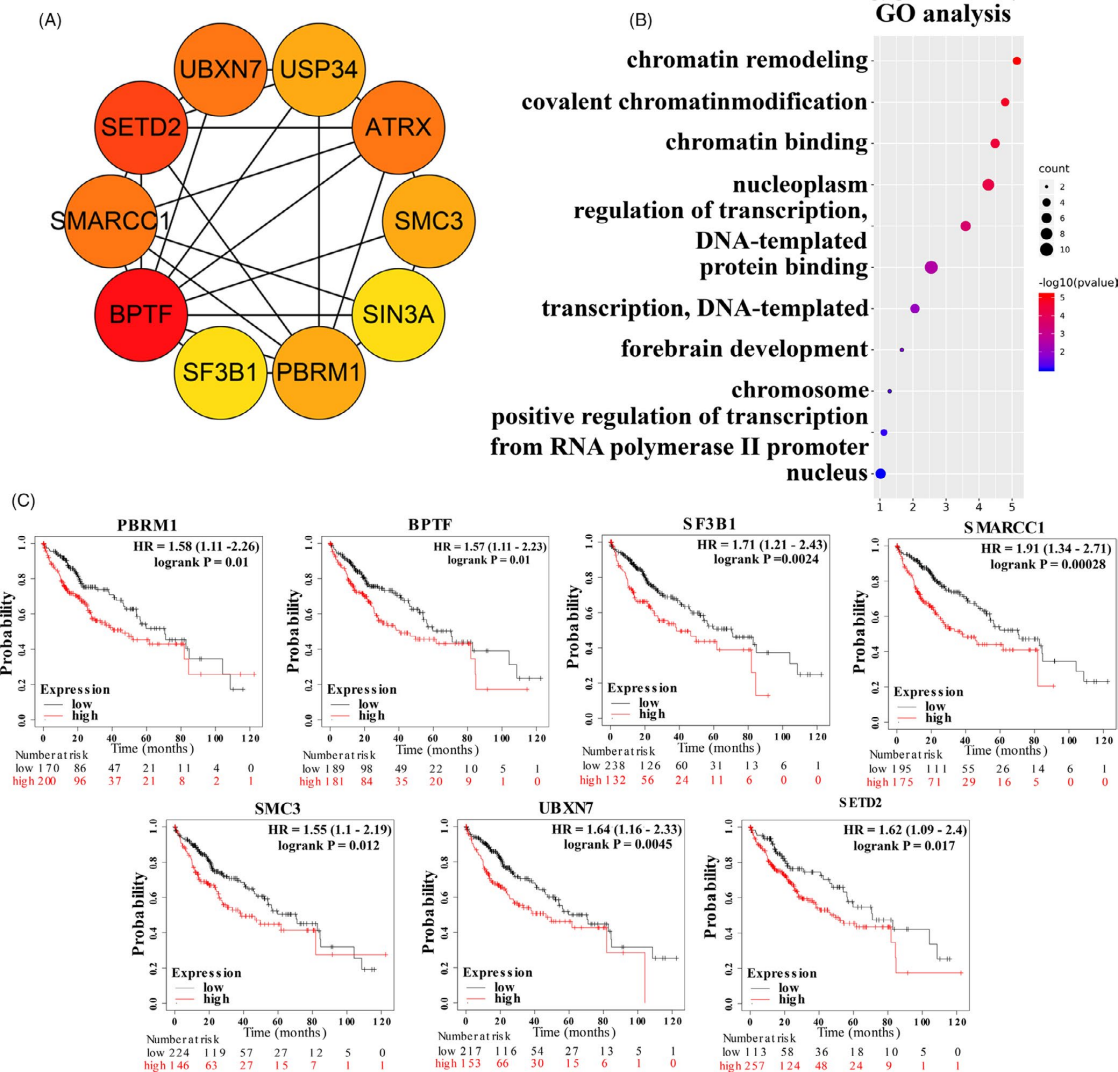
Hub gene analysis. A, The interaction network of the top 10 hub genes. B, GO analysis of the top 10 hub genes. C, The prognostic values of seven hub genes (*BPTF*,* SETD2*,* SMARCC1*,* UBXN7*,* SMC3*,* PBRM1* and *SF3B1*)

First, the most important clusters in the PPI network were identified using MCODE (Figure 8B, shown in yellow). The top ten hub genes of the network were identified using cytoHubba (ranked by degree) (Figure 9A). GO analysis results showed that biological processes, such as chromatin remodelling, covalent chromatin modification and chromatin binding, were significantly affected and enriched (Figure 9B). Then, the prognostic value of the hub genes was evaluated in Kaplan‐Meier Plotter. Among these ten genes, the high expression of seven genes was significantly related to poor OS (*BPTF*,* SETD2*,* SMARCC1*,* UBXN7*,* SMC3*,* PBRM1* and *SF3B1*) (Figure 9C), while the other three genes showed no significance (*ATRX*,* SIN3A* and *USP34*) (Figure S3).

### 3.8 Validation of the interaction between USP1 and WDR48 in HCC cell lines

**Figure 10:**
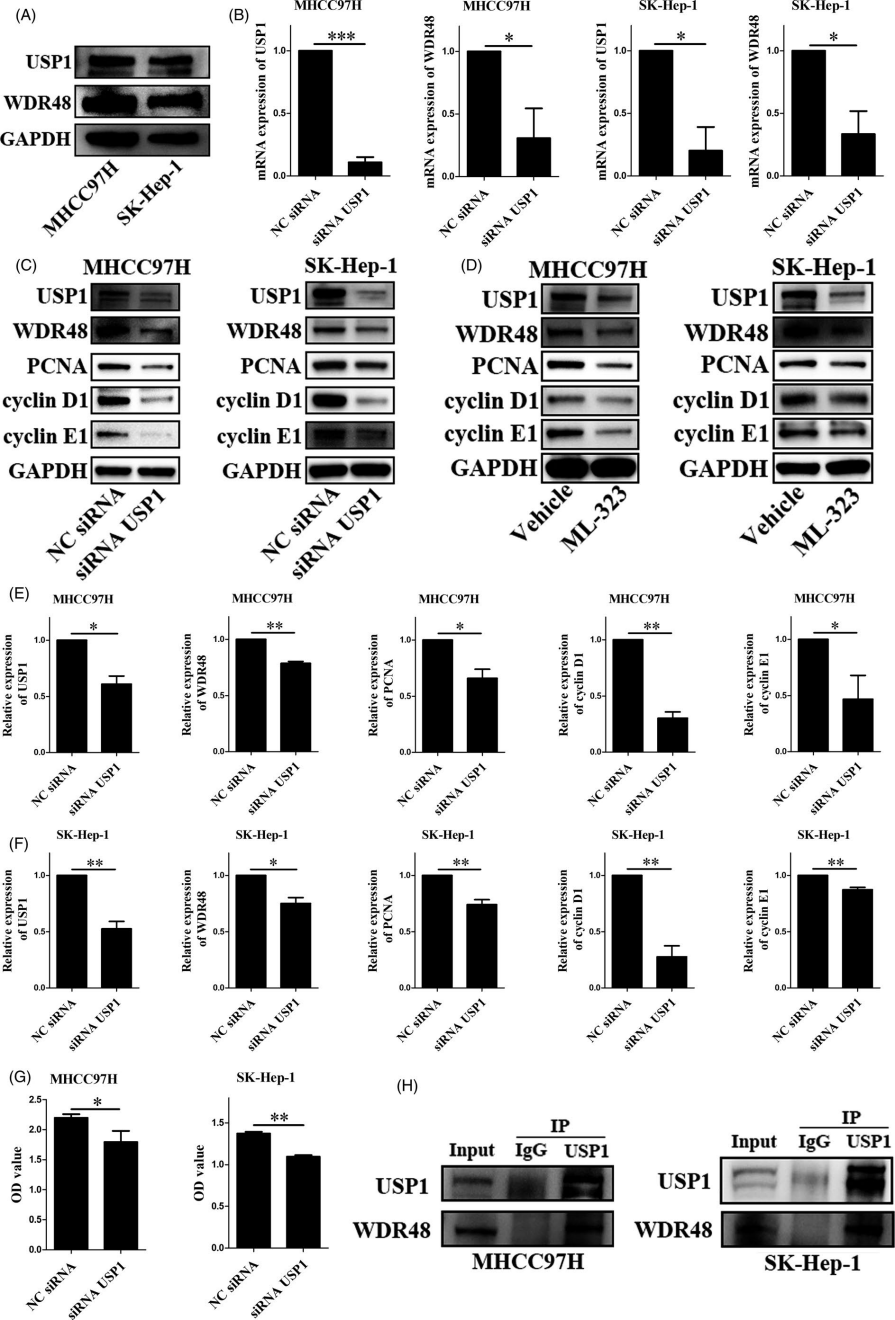
Functional analysis of USP1 and WDR48 in MHCC97H and SK‐Hep‐1 cells. A, Protein expression of USP1 and WDR48 in MHCC97H and SK‐Hep‐1 cells. B, mRNA expression of USP1 and WDR48 after siRNA‐USP1 or non‐targeting siRNA (NC‐siRNA) transfection. *P < .05, **P < .01, ***P < .001. C,E,F, Protein expression of USP1 and WDR48 after siRNA‐USP1 or NC‐siRNA transfection. *P < .05, **P < .01, ***P < .001. D, Protein expression of USP1 and WDR48 after vehicle or ML‐323 treatment. G, Cell viability measured by cell counting kit‐8 after siRNA‐USP1 or NC‐siRNA transfection. *P < .05, **P < .01, ***P < .001. H, Co‐immunoprecipitation results of USP1 and WDR48 in MHCC97H and SK‐Hep‐1 cells

First, we detected the expression of USP1 and WDR48 using Western blotting. We found that protein levels of USP1 and WDR48 were highly expressed in HCC cell lines, both in MHCC97H and in SK‐Hep‐1 (Figure 10A). Then, we knocked down USP1 in these cell lines using siRNA targeting USP1. After siRNA‐USP1 transfection, USP1 was significantly down‐regulated compared with the NC‐siRNA transfected cells, both at protein and mRNA levels (Figure 10B,C,E,F). Moreover, WDR48 was significantly reduced by USP1 knockdown in MHCC97H and SK‐Hep‐1 cells (Figure 10B,C,E,F). These results were further validated by a specific USP1 inhibitor ML‐323. After 50 μmol/L ML‐323 treatments for 24 hours, both USP1 and WDR48 were down‐regulated in MHCC97H and SK‐Hep‐1 cells (Figure 10D). In addition, we confirmed the interaction between USP1 and WDR48 in HCC cell lines using co‐immunoprecipitation, and the results showed that USP1 interacted with WDR48 in MHCC97H and SK‐Hep‐1 cells (Figure 10H).

### 3.9 SiRNA‐USP1 transfection or ML‐323 treatment decreased the proliferation of HCC cells

As mentioned above, the Figure 8F showed that the coexpressed genes were mainly involved in the cell cycle. Thus, we explored the role of *USP1* in the proliferation of HCC cells in the following experiments. SiRNA‐USP1 was transfected into MHCC97H and SK‐Hep‐1 cells. First, CCK‐8 assays were conducted to evaluate the cell viability and the cell proliferation ability. The results indicated that USP1 knockdown significantly inhibited the proliferation of MHCC97H and SK‐Hep‐1 cells (Figure 10G). Proliferating cell nuclear antigen (PCNA), one of the cellular targets of USP1/WDR48,^44^ was significantly decreased after USP1 knockdown. Moreover, USP1 knockdown also decreased the expression of cyclin D1 and cyclin E1, thereby inhibited liver cell growth via cell cycle arrest^45^ (Figure 10C,E,F). Moreover, ML‐323 treatment also decreased the protein expression of PCNA, cyclin D1 and cyclin E1. Thus, we made a conclusion that targeting USP1 could reduce the proliferation of HCC cells.

## 4 DISCUSSION

Currently, HCC remains a worldwide health problem with poor prognosis and high death rates. Late diagnosis, metastasis and quick progression are the main causes of cancer‐induced death in patients with HCC.^46^ If the patients are diagnosed at an early stage and given effective therapies, their survival may significantly improve.^47^ Thus, on the one hand, identifying hallmarks for the early diagnosis and tumour progression of HCC is urgently needed.^48^ On the other hand, it is crucial to find novel therapeutic targets and develop new therapeutic strategies.^49^ In recent years, with the development of sequencing and omics, we can further understand the underlying mechanisms of HCC.^50^

As key regulators of ubiquitination, deubiquitinating family enzymes play important roles in tumour diseases.^51^, ^52^ In recent years, our understanding of deubiquitinases has made great progress, especially in HCC. The effect of deubiquitinases depends mainly on their substrate, either to promote or suppress tumour progression.^53^ USP4 interacts with cyclophilin A and TGF‐β receptor type I and promotes the progression and metastasis of HCC.^54^ USP5 stabilizes SLUG and promotes epithelial‐mesenchymal transition.^55^ USP7 regulates the Hippo pathway by deubiquitinating Yorkie and predicts the prognosis of HCC.^56^, ^57^ USP10 maintains the activity of Yes‐associated protein (YAP) and transcriptional coactivator with PDZ‐binding motif (TAZ), stabilizes Smad4 protein and then promotes the proliferation of HCC.^58^, ^59^ However, to our knowledge, few studies have investigated the role of *USP1* in liver cancer. Considering the importance of *USP1* in regulating DNA repair, it has long been considered a potential therapeutic target for tumours.^15^ The activity of *USP1* may reflect the treatment response, which may help identify patients with chemoresistance.^14^ However, there are several questions that we could not find the answers in previous studies. First, what alterations of *USP1* occur in HCC: mutational alterations, expressional alterations or both? Second, do these alterations have clinical significance? Do these alterations have an association with the prognosis of patients? Third, what is the underlying mechanism of *USP1* in HCC? For these reasons, we systemically analysed the role of *USP1* in HCC using a set of informatics tools. We believe our findings in this study could at least partially explain the abovementioned questions.

In this study, we confirmed the higher expression of *USP1* in HCC than in normal tissues. High expression of *USP1* showed clinical significance and was associated with unfavourable survival in HCC patients. These results suggest that *USP1* is a potential therapeutic target in HCC. However, the promoter methylation level of *USP1* did not significantly change between HCC and normal tissues, which meant that the alteration of *USP1* expression was not due to this type of posttranslational modification. In present, the mechanisms underlying *USP1* overexpression in human cancer is still not fully understood. In general, *USP1* is phosphorylated by CDK1 at S313, and then binding with WDR48 for activation.^60^ As previously reported, USP1 could be degraded by APC/C Cdh1 during G1 phase. Moreover, calpain inhibits Cdh1, and thus inhibits USP1 degradation.^44^ As we know, Cdh1 is generally accepted as a tumour suppressor,^61^ USP1 overexpression in HCC tissues may partially due to the dysregulation of Cdh1. However, more studies are needed to fully understand the mechanisms underlying USP1 overexpression in HCC. In addition to expressional alterations in HCC, we also found several mutational alterations of *USP1*, mainly missense substitutions. However, the mutation frequency was relatively low (only 0.3%), and we failed to find an association between these mutations and prognosis. More data are needed to clarify the clinical significance of these mutations. Then, another issue was raised: why did the high expression of *USP1* correlate with the unfavourable survival of patients? Here, we found that the high expression of *USP1* was positively correlated with immune infiltration. This finding suggests that *USP1* plays a critical role in immune infiltration during HCC development. To our knowledge, although USPs have been reported to be involved in the regulation of the immune response, we are the first to analyse the association of *USP1* and immune infiltration in HCC.^62^ In addition, we also tried to explain the underlying mechanisms of *USP1* in HCC. As reported, *USP1* alone had low deubiquitinase activity, and its activity was significantly promoted when forming a complex with its binding partner *WDR48*.^63^ *USP1‐WDR48* mainly works by maintaining the activity of their substrates. Yu et al reported that the *USP1‐WDR48* complex stabilized TANK‐binding kinase 1 by moving its K48‐linked polyubiquitination, and this process could be attenuated using the inhibitor ML‐323.^63^ Intriguingly, we found that *WDR48* was also overexpressed in HCC tissues compared with normal tissues and was associated with poor prognosis in patients. Its expression showed a positive correlation with *USP1*. Moreover, we confirmed the interaction of USP1‐WDR48 in HCC cells using co‐immunoprecipitation. This finding indicated that *USP1* and *WDR48* were closely linked and may function together. Hence, we hypothesized that the *USP1‐WDR48* complex played critical roles in HCC by stabilizing the activity of their substrates. To identify these important substrates, we focused on the differentially expressed genes in HCC that were positively correlated with both *USP1* and *WDR48* and identified 98 differentially expressed genes. According to the GO analysis results, these genes were mainly enriched in the following processes: transcription, regulation of transcription, covalent chromatin modification, etc The KEGG enrichment analysis results showed that these genes were mainly enriched in the cell cycle, aldosterone synthesis and secretion and oestrogen signalling pathways. More importantly, we identified 10 hub genes among these genes (*BPTF*,* SETD2*,* SMARCC1*,* UBXN7*,* SMC3*,* PBRM1*,* SF3B1*,* ATRX*,* SIN3A* and *USP34*). Of these, seven genes (*BPTF*,* SETD2*,* SMARCC1*,* UBXN7*,* SMC3*,* PBRM1* and *SF3B1*) showed prognostic value in HCC patients. This finding suggested that the *USP1‐WDR48* complex played a tumour‐promoting role in HCC by stabilizing and deubiquitinating these hub genes. As mentioned above, the function of USP1 is depended on its cofactor WDR48, which give us the opportunity to develop specific therapeutic strategies. In the past years, several agents aimed at USP1 have been reported, such as pimozide and ML‐323.^64^, ^65^ These agents inhibit the activity of USP1‐WDR48 complex in a non‐competitive manner.^66^ In the present study, we demonstrated that siRNA‐USP1 transfection or ML‐323 treatment decreased the proliferation of HCC cells. USP1 knockdown or ML323 treatment reduced the expression of PCNA, cyclin D1 and cyclin E1, which meant that targeting USP1 could decrease the proliferation of HCC cells via cell cycle arrest.

Nevertheless, there are several limitations to this study. First, most of the data included for analysis were mined from public databases and validated in in vitro experiments; however, some of the results may need to be further validated in the future study. Second, in addition to the expressional alteration of *USP1*, we should also pay attention to the alteration of its activity. Nevertheless, we can conclude that *USP1* is a promising therapeutic target in the treatment of HCC.

## 5 CONCLUSIONS

In this study, we found that *USP1* was highly expressed in HCC and predicted the poor prognosis of patients, suggesting it as a promising therapeutic target for HCC *USP1* was positively correlated with immune infiltration. *USP1* and its cofactor *WDR48* are involved in the tumour progression of HCC by deubiquitinating and stabilizing their substrates. These potential substrate genes were mainly enriched in the cell cycle, aldosterone synthesis and secretion and oestrogen signalling pathways. Targeting USP1 could decrease the proliferation of HCC cells via cell cycle arrest.

## CONFLICT OF INTEREST

None.

## AUTHOR CONTRIBUTION

LJ Li conceived and designed this study. YL Zhao and C Xue searched the databases and analysed the data. YL Zhao drafted the manuscript. C Xue prepared the figures and tables. ZY Xie and XX Ouyang reviewed and revised the manuscript. All authors read and approved the final manuscript for publication.

## ETHICAL APPROVAL

The study was conducted based on data in the public databases, there is no ethical statement to be declared.

## Supporting information

Fig S1

Fig S2

Fig S3

## Data Availability

All of the data involved in this study are available in the public databases which are listed in the Materials and methods section.
