# Supplementary figures and images for: Comprehensive analysis of ubiquitin‐specific protease 1 reveals its importance in hepatocellular carcinoma
Source: Cell Prolif. 2020 Sep 19;53(10):e12908. doi: 10.1111/cpr.12908 (PMC7574869; doi:10.1111/cpr.12908)

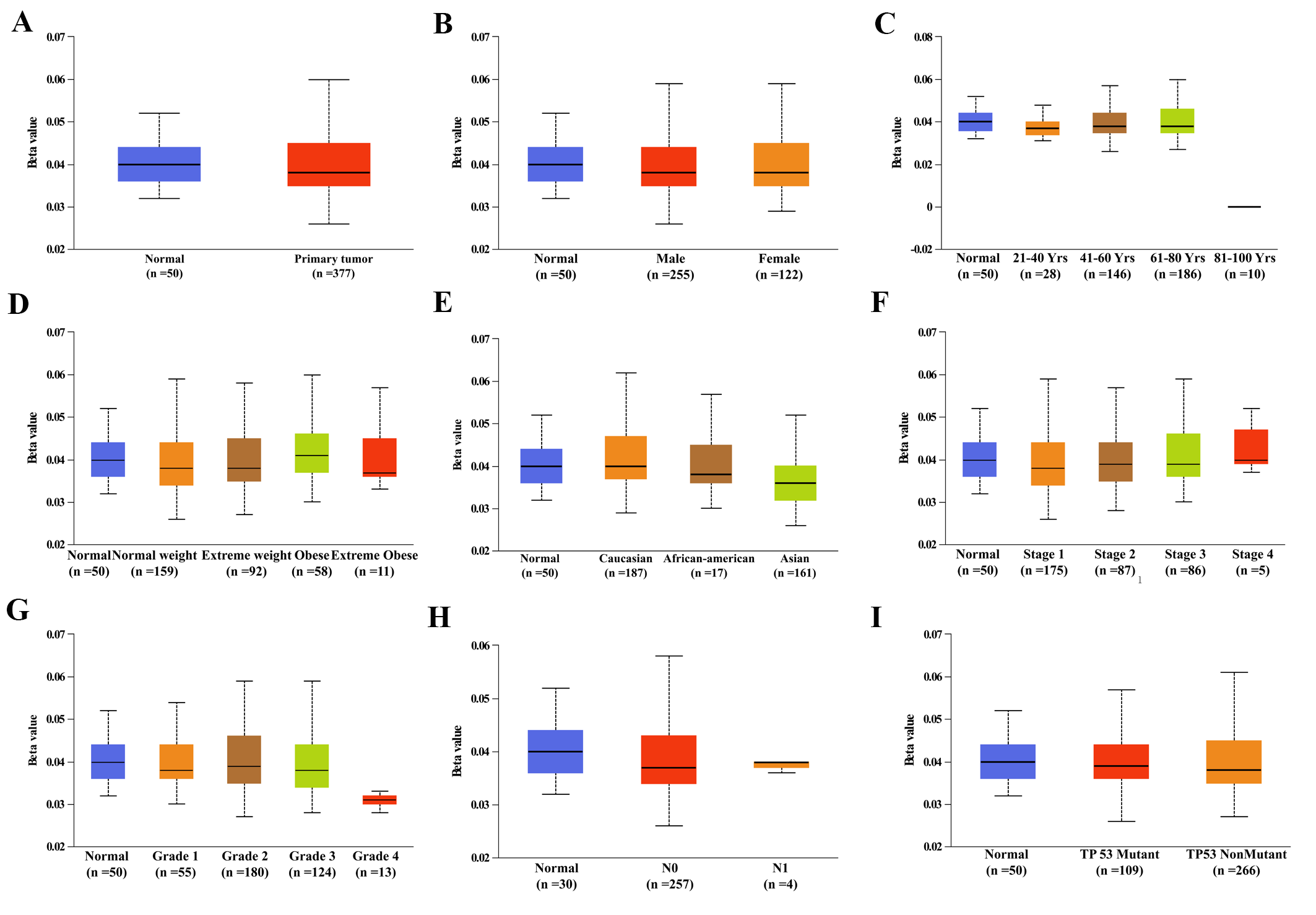

Supplement: Supplementary file 1 — Fig S1 [file CPR-53-e12908-s001.tif]

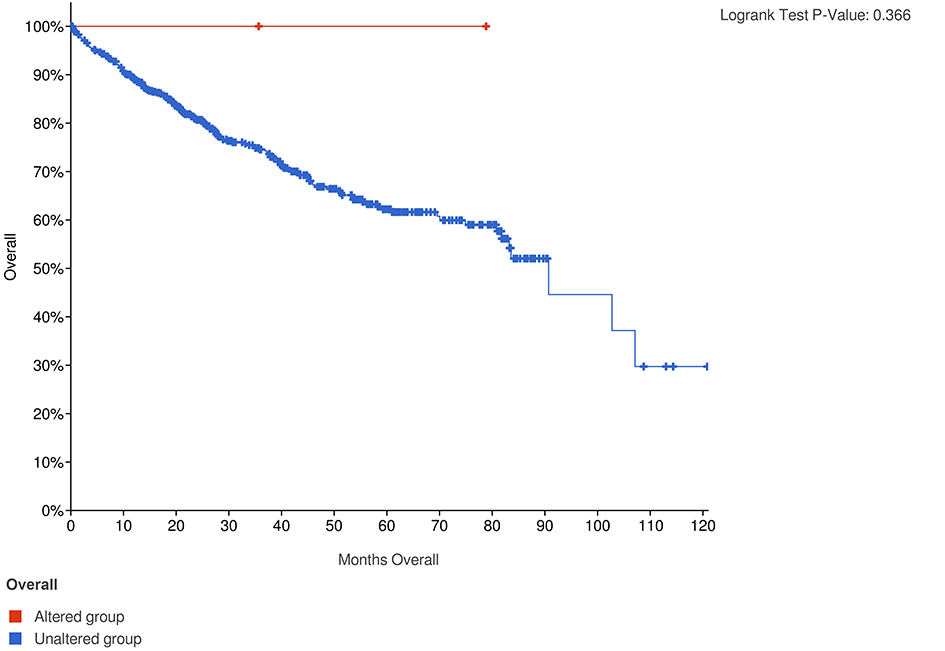

Supplement: Supplementary file 2 — Fig S2 [file CPR-53-e12908-s002.tif]

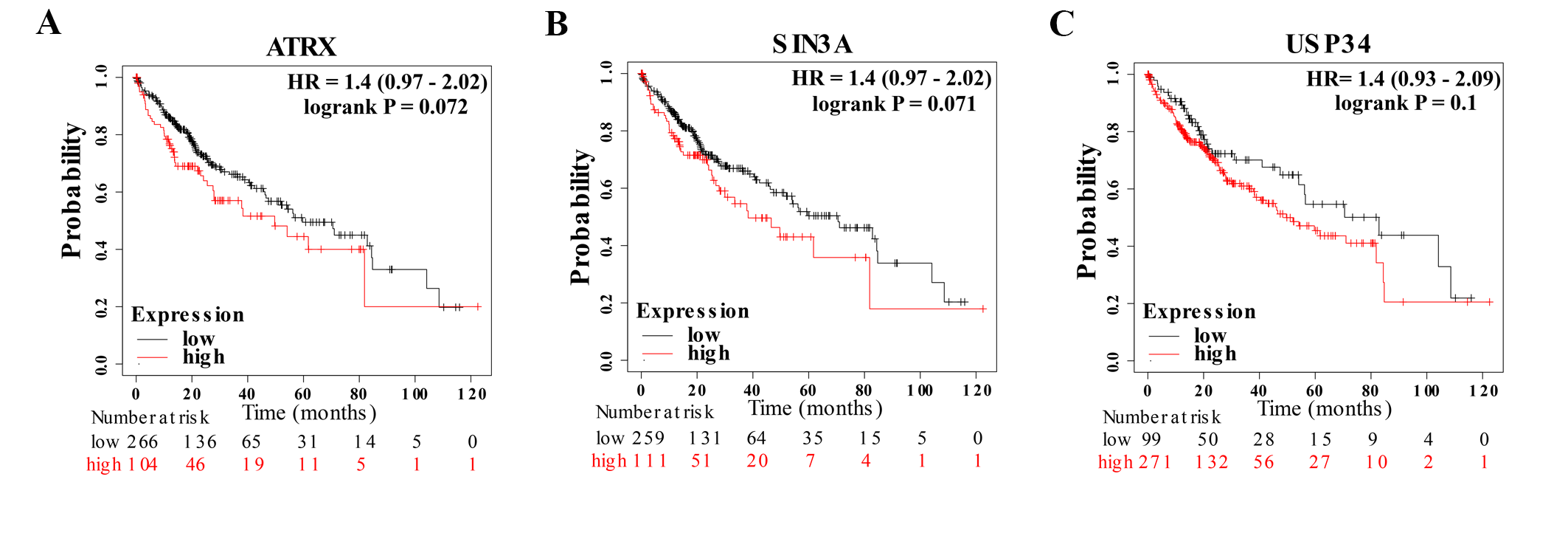

Supplement: Supplementary file 3 — Fig S3 [file CPR-53-e12908-s003.tif]
